# ST-HADP: Spatio-Temporal hierarchical attention diffusion policy for long-horizon generalizable bimanual visuomotor imitation

**DOI:** 10.3389/fnbot.2026.1846433

**Published:** 2026-06-08

**Authors:** Xukun Liu, Fengjuan Xie, Shibo Liu, Xu Sun, Zhenyu Liu, Guangning Li, Shenggang Wei

**Affiliations:** Northwest Institute of Mechanical and Electrical Engineering, Xianyang, Shaanxi, China

**Keywords:** 3D visuomotor policy, bimanual manipulation, hierarchical diffusion, imitation learning, Spatio-Temporal hierarchical attention

## Abstract

**Introduction:**

Dual-arm robotic manipulation presents fundamental challenges in coordinating spatially shared perception and temporally extended behaviors under limited demonstration settings. Existing diffusion-based visuomotor policies rely on flat temporal horizons and globally pooled visual features, which fail to capture the structured nature of bimanual collaboration.

**Methods:**

We propose the Spatio-Temporal Hierarchical Attention Diffusion Policy (ST-HADP), a framework that extends 3D diffusion policies through explicit spatial and temporal structuring. ST-HADP introduces a Spatial Attention Module that learns arm-specific focus over task-relevant 3D regions, enabling dynamic and coordinated spatial reasoning. It further incorporates a Temporal Abstraction Module that models action sequences across multiple timescales via hierarchical latent variables, facilitating coarse-to-fine action generation aligned with the natural progression of long-horizon tasks. These components are jointly optimized with a multi-objective loss function that integrates attention regularization and temporal consistency, promoting spatially focused and temporally smooth coordination.

**Results:**

We evaluate ST-HADP on the RoboTwin 2.0 platform across six dual-arm robot configurations with diverse morphologies and tasks. Using only 50 automatically generated expert demonstrations, our method consistently outperforms baseline policies, achieving higher success rates with modest additional computational overhead.

**Discussion:**

The results demonstrate that explicit spatial and temporal structuring enables effective dual-arm coordination under limited demonstration settings. ST-HADP provides a generalizable framework for bimanual manipulation, suggesting that hierarchical attention mechanisms offer a promising direction for sample-efficient learning of coordinated multi-arm behaviors.

## 1 Introduction

Dual-arm robotic manipulation has emerged as a critical capability for complex real-world tasks. Unlike single-arm systems, bimanual setups require coordinated perception and action across two agents operating in a shared physical space, necessitating policies that can reason over spatial dependencies, temporally extended behaviors, and dynamic role allocation. Despite recent progress in visuomotor policy learning, existing methods often fall short in addressing the unique challenges of dual-arm coordination, particularly under limited demonstration data and in the presence of task-phase variability.

Diffusion-based policies have shown promise in modeling multimodal action distributions and have been successfully applied to a range of robotic manipulation tasks. However, most existing approaches rely on flat temporal horizons and globally pooled visual features, which fail to capture the structured nature of long-horizon bimanual tasks. These methods treat the entire scene uniformly, overlooking the need for arm-specific spatial attention and temporal abstraction, which fundamentally limits their capacity to achieve robust spatial generalization, maintain coherent cross-arm coordination, and exhibit consistent behavioral patterns across varying task phases.

To address these limitations, we propose the Spatio-Temporal Hierarchical Attention Diffusion Policy (ST-HADP), a novel framework that extends 3D diffusion policies by introducing explicit spatial and temporal structuring for dual-arm manipulation. Our method enhances shared 3D scene representations with a spatial attention module that learns arm-specific focus over task-relevant regions, enabling dynamic and coordinated spatial reasoning. It further incorporates a temporal abstraction module that models action sequences across multiple timescales through hierarchical latent variables, facilitating coarse-to-fine action generation that aligns with the natural progression of long-horizon tasks. These components are jointly optimized with a multi-objective loss function that integrates attention regularization and temporal consistency, encouraging spatially focused and temporally smooth coordination. This unified design enables ST-HADP to robustly capture both the spatial specialization and temporal structure inherent in bimanual manipulation, leading to strong performance under limited demonstration data.

We evaluate ST-HADP on the RoboTwin 2.0 platform, a comprehensive benchmark for bimanual manipulation that features diverse robot morphologies—including asymmetric arm configurations—and extensive domain-randomized tasks with variations in object appearance and initial poses parameters. Using 50 automatically generated expert demonstrations, our method consistently outperforms 3D Diffusion Policy (DP3) across six dual-arm configurations, achieving higher success rates with minimal computational overhead.

## 2 Related work

Recent advances in diffusion models have reshaped generative modeling and robot visuomotor learning. Denoising Diffusion Probabilistic Models (DDPM) (Ho et al., 2020) and score-based formulations (Song et al., 2020) established stable likelihood-based training with strong multimodal expressiveness, while accelerated samplers like DDIM (Song et al., 2020) improved inference efficiency. Originally developed for high-fidelity image synthesis (Rombach et al., 2022), diffusion models were extended to sequential decision-making, including offline reinforcement learning (Wang et al., 2022) and trajectory optimization (Janner et al., 2022). Building on this foundation, Diffusion Policy (Chi et al., 2025) formulated visuomotor control as conditional action denoising, demonstrating superior robustness over autoregressive behavior cloning. Subsequent works explored goal-conditioned diffusion (Reuss et al., 2023), keypose-guided generation (Fan et al., 2025), consistency-accelerated sampling (Prasad et al., 2024), and reward-conditioned modeling (Huang et al., 2024), establishing diffusion as a flexible alternative to deterministic policies.

Despite strong multimodal capabilities, most diffusion-based visuomotor policies operate with flat temporal horizons and globally pooled features. While effective for single-arm tasks, such designs fail to model structured behavioral phases or coordinated spatial specialization—properties essential for dual-arm and long-horizon collaboration.

In parallel, visual imitation learning has evolved toward geometry-aware representations. Benchmarks like MetaWorld (Yu et al., 2020) and Adroit dexterous manipulation environments (Jian et al., 2026) enabled systematic policy comparison, while Implicit Behavioral Cloning (IBC) (Florence et al., 2022), recurrent BC (Lynch et al., 2020), and representation-centric analyses (Jiang et al., 2024) highlighted stability under limited demonstrations. Transformer-based policies like ACT (Zhao et al., 2023) improved temporal abstraction via action chunking but remained dependent on 2D visual tokens. Purely image-conditioned policies suffer from viewpoint sensitivity, occlusion, and limited spatial extrapolation, underscoring the need for explicit 3D scene representations that enable task-aware spatial reasoning beyond pixel-level features.

Recent works have incorporated 3D representations to overcome spatial reasoning bottlenecks. Point-based encoders [PointNet (Qi et al., 2017a), PointNet++ (Qi et al., 2017b), Point Transformer (Zhao et al., 2021), PointNeXt (Qian et al., 2022)] provide permutation-invariant geometric feature extraction. Manipulation-oriented architectures [PerAct2 (Grotz et al., 2024), RVT (Goyal et al., 2023), ACT3D (Wei et al., 2025), Perceiver-actor (Shridhar et al., 2023), NeRFuser (Yan et al., 2024)] demonstrate that explicit geometry modeling enhances cross-view generalization. Diffusion-based 3D control emerged with 3D Diffuser Actor (Ke et al., 2024) and 3D Diffusion Policy (DP3) (Ze et al., 2024), integrating point cloud conditioning into action denoising. However, DP3’s global pooling strategy treats all spatial regions uniformly, failing to disentangle arm-specific focus or dynamically reallocate attention as task phases evolve—limiting its ability to model cooperative perception and action in dual-arm manipulation settings.

Dexterous and long-horizon manipulation amplify these challenges due to high dimensionality and error accumulation. Large-scale RL systems [OpenAI Dactyl (Akkaya et al., 2019), Orbit (Mittal et al., 2023)], enabled by parallel simulators like Isaac Gym (Makoviychuk et al., 2021), demonstrate high-DoF skill acquisition with sufficient data. Imitation-based approaches [Cyberdemo (Wang et al., 2024), DexMV (Qin et al., 2022), vision-based retargeting (Handa et al., 2020)] reduce sample complexity but often depend on structured intermediate representations. Recent work on articulated and deformable objects [DexArt (Bao et al., 2023), DexDeform (Li et al., 2023)] highlights the importance of robust spatial feature extraction under limited data. While diffusion-based policies help mitigate multimodal ambiguity in high-dimensional control settings, their stability remains highly sensitive to conditioning quality, feature redundancy, and the propagation of noise across denoising steps—especially in tasks involving sequential and coordinated manipulation. These vulnerabilities highlight the need for structured temporal abstraction and spatially coordinated conditioning to ensure stable performance in long-horizon, dual-arm manipulation tasks.

Comprehensive evaluation platforms are essential for assessing generalization and coordination. Simulation environments like MuJoCo (Todorov et al., 2012), Sapien (Xiang et al., 2020), and PlasticineLab (Huang et al., 2021) provide diverse physics settings but face limitations in complex long-horizon tasks. Multi-task benchmarks like CALVIN (Mees et al., 2022) focus on compositional reasoning but do not fully capture coordinated manipulation across varying environments. In contrast, RoboTwin 2. 0 (Chen et al., 2025) offers a comprehensive platform for dual-arm and collaborative tasks, incorporating coordinated perception-action coupling, shared-scene reasoning, and high-dimensional bimanual control—making it ideal for evaluating spatial specialization and cooperative visuomotor policies in complex, dynamic environments.

## 3 Method

We consider the problem of learning collaborative visuomotor policies for dual-arm robotic systems from a small set of expert demonstrations. Given two robot arms that must coordinate to complete a sequential manipulation task, we aim to learn a policy $\pi :O\mapsto A_{1}\times A_{2}$ that maps shared visual observations oεO to joint actions $(a_{1},a_{2})\varepsilon A_{1}\times A_{2}$ for both arms. Beyond faithfully reproducing collaborative manipulation skills, the learned policy must generalize robustly to previously unseen scenarios, including variations in object position, orientation, and visual appearance.

Motivated by these challenges in coordination and generalization, we propose the Spatio-Temporal Hierarchical Attention Diffusion Policy (ST-HADP), extending the 3D Diffusion Policy framework with three key innovations:

1. A Spatial Attention Module (SAM) for task-aware 3D feature weighting.
2. A Temporal Abstraction Module (TAM) for coarse-to-fine hierarchical action generation.
3. A multi-component loss function with temporal consistency and attention regularization.

**Figure 1:**
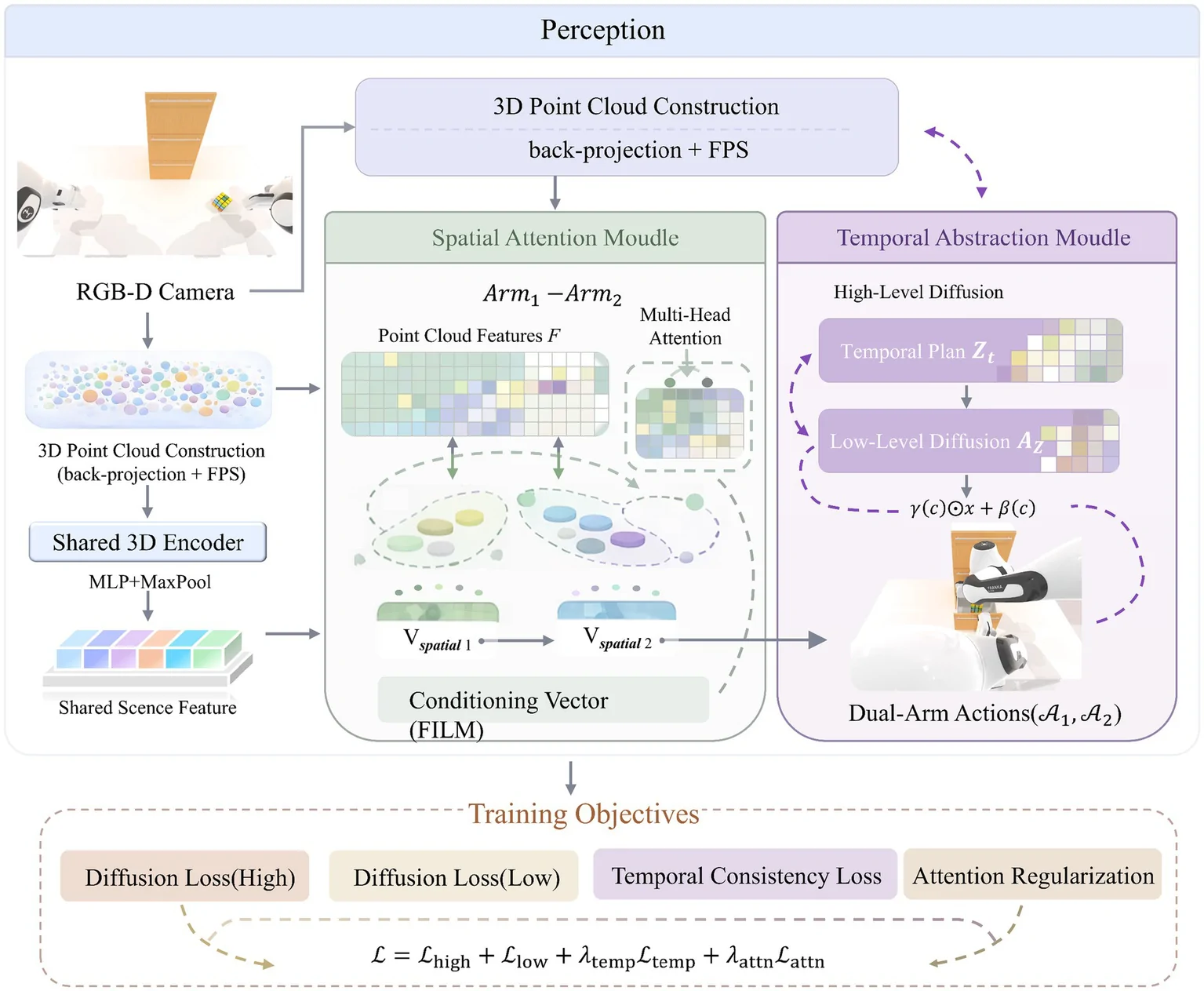
Overview of ST-HADP architecture.

An overview of ST-HADP is presented in Figure 1.

A Problem formulation

We formalize the dual-arm manipulation task as a Markov Decision Process (MDP) (Equation 1):

M=(O,A,p,r) (1)

At each timestep t , the agent receives a shared RGB-D observation captured by a single fixed camera covering the entire workspace, including both robot arms, the cabinet, and the target object (Equation 2).

$$o_{t}\in {\mathbb{R}}^{H\times W\times 4}$$ (2)

The action space is factorized as (Equation 3):

$$\begin{array}{c} A_{t}=(A_{1},A_{2}) \end{array}$$ (3)

The mapping from $A_{1}$ and $A_{2}$ to the door-opening and object-placing arms is not predetermined. Instead, role assignment is determined by a simple distance-based heuristic during execution: the arm whose end-effector is closer to the cabinet handle at the start of the task is assigned the door-opening role, while the other arm is assigned the object-placing role. This heuristic leverages the spatial observation provided by the point cloud and is computed at the first timestep of each episode.

Given a dataset of expert demonstrations (Equation 4):

$$\begin{array}{c} D={\left\{\tau_{i}\right\}}_{i=1}^{N},\tau_{i}={\left\{(o_{t},A_{t})\right\}}_{t=1}^{T_{i}} \end{array}$$ (4)

Our objective is to learn a policy that maximizes trajectory likelihood while enabling generalization to novel object positions, cabinet configurations, and visual appearances.

B Shared 3D visual perception

A defining characteristic of dual-arm manipulation is that both manipulators operate within a physically shared 3D workspace. Effective coordination therefore requires modeling both arm–object relationships and cross-arm geometric interactions.

Purely 2D feature extraction from RGB images is insufficient for capturing such spatial dependencies, as it lacks explicit depth and metric structure. We therefore construct a unified 3D scene representation that explicitly encodes the shared workspace geometry.

Point cloud construction. At each timestep t , the depth map $D_{t}\in {\mathbb{R}}^{H\times W}$ is back-projected into a metric point cloud. For each pixel (u,v) with depth value d , the corresponding 3D point is computed as (Equation 5):

$$\begin{array}{c} p=E^{-1}(d\cdot K^{-1}{\left[u,v,1\right]}^{T}) \end{array}$$ (5)

where K and E are camera intrinsics and extrinsics.

Applying this transformation to all pixels yields a raw point cloud (Equation 6):

$$\begin{array}{c} P_{t}^{\mathrm{raw}}={\left\{p_{i}\right\}}_{i=1}^{\mathrm{HW}} \end{array}$$ (6)

Which contains points from both robot arms, the cabinet, the manipulated object, and the surrounding environment.

Spatial cropping and downsampling. To focus on task-relevant regions and reduce computational overhead, the raw point cloud is confined to a predefined bounding region $ℬ\subset {\mathbb{R}}^{3}\;$ and subsequently downsampled to a fixed size via farthest point sampling (FPS) (Equation 7).

$$\begin{array}{c} P_{t}={\left\{p_{i}\right\}}_{i=1}^{M} \end{array}$$ (7)

FPS ensures uniform coverage of the 3D space and reduces sampling randomness compared to uniform sampling.

Shared 3D encoding. The downsampled point cloud is encoded into a compact shared representation using a lightweight MLP-based encoder. Each point is processed independently by a shared multi-layer perceptron (Equation 8):

$$\begin{array}{c} f_{i}={\mathrm{MLP}}_{\text{shared}}(p_{i}),f_{i}\in {\mathbb{R}}^{256} \end{array}$$ (8)

Permutation invariance is achieved via max pooling (Equation 9):

$$\begin{array}{c} f_{\text{pool}}=\overset{M}{\max_{i=1}}\;f_{i},f_{\text{pool}}\in {\mathbb{R}}^{256} \end{array}$$ (9)

The pooled feature is then projected to a low-dimensional embedding (Equation 10):

$$\begin{array}{c} v=\text{Proj}(f_{\text{pool}}),v\in {\mathbb{R}}^{64} \end{array}$$ (10)

${\mathrm{MLP}}_{\text{shared}}$ consists of three linear layers with interleaved LayerNorm and ReLU activations, and the projection head further compresses the global feature (Equation 11).

$$\begin{array}{c} {\mathrm{MLP}}_{\text{shared}}(x)=\text{ReLU}(\mathrm{LN}(W_{3}\cdot \text{ReLU}(\mathrm{LN}(W_{2}\cdot \text{ReLU}(\mathrm{LN}(W_{1}x+b_{1}))+b_{2}))+b_{3})) \end{array}$$ (11)

The resulting shared feature $v\in {\mathbb{R}}^{64}$ encodes the overall 3D scene geometry and provides a common perceptual basis for both arms’ policies.

C Spatial attention module (SAM)

Dual-arm manipulation requires spatial specialization. Each arm must focus on distinct yet evolving scene regions. The Spatial Attention Module (SAM) addresses this by learning dynamic, arm-specific attention masks over the shared point cloud.

Multi-head spatial attention. For each arm j∈{1,2} , SAM computes a multi-head attention mask that weights each point’s contribution to the arm’s feature representation (Equation 12):

$$\begin{array}{c} \alpha^{j}=\text{softmax}(\frac{(W_{q}^{j}F){\left(W_{k}^{j}F\right)}^{T}}{\sqrt{d_{k}}})\in {\mathbb{R}}^{M\times M} \end{array}$$ (12)

Where $W_{q}^{j},W_{k}^{j}\in {\mathbb{R}}^{d_{k}\times 256}$ are learnable query and key projections for arm j .

Attended feature aggregation. The attended features for each arm are computed by applying the attention weights to the value-projected point features (Equation 13):

$$\begin{array}{c} F_{\text{attn}}^{j}=\alpha^{j}(FW_{v}^{j})\in {\mathbb{R}}^{M\times 256} \end{array}$$ (13)

Where $W_{v}^{j}\in {\mathbb{R}}^{256\times 256}$ is a value projection.

Global arm-specific features are obtained via max pooling (Equation 14):

$$\begin{array}{c} f_{\text{pool}}^{j}=\overset{M}{\max_{i=1}}{\left[F_{\text{attn}}^{j}\right]}_{i}\in {\mathbb{R}}^{256} \end{array}$$ (14)

Which are projected to (Equation 15):

$$\begin{array}{c} V_{\text{spat}i\mathrm{al}}^{j}=\mathrm{Pro}j_{\text{spatial}}(f_{\text{pool}}^{j}),v_{\text{spat}i\mathrm{al}}^{j}\in {\mathbb{R}}^{64} \end{array}$$ (15)

These features provide task-adaptive spatial representations for each arm.

Attention regularization loss. To encourage the attention masks to focus on semantically meaningful regions and avoid diffuse attention patterns, we introduce an attention regularization loss ${ℒ}_{\text{attn}}$ . A critical aspect of this regularization is the identification of task-irrelevant points (e.g., table surface, background structures, or the non-grasping parts of the robot arms) that the policy should learn to ignore.

To identify task-irrelevant points, we obtain binary masks ${ℳ}^{j}$ for each arm j using per-point semantic annotations provided by the simulation environment. Points belonging to predefined irrelevant categories (e.g., table surface, background structures, non-grasping parts of the robot arms) constitute the set ${ℐ}^{j}$ . While this introduces privileged information during simulation training, it provides a clean learning signal to validate the effectiveness of our attention regularization mechanism.

For real-world deployment, the reliance on simulator labels can be eliminated by employing off-the-shelf visual foundation models in a zero-shot manner to segment task-relevant regions from the point cloud. These models produce semantically meaningful masks without requiring manual annotation or privileged information. Furthermore, a fully self-supervised alternative, such as entropy-based attention weighting or spatial–temporal variance-based dynamic region suppression, could be explored to further reduce dependency on explicit labels. We consider systematic evaluation of these transfer strategies as important future work and retain privileged labels in this study to ensure stable training and clear ablation.

Justification of the 64-dimensional spatial embedding. The choice of a 64-dimensional bottleneck for $v_{\text{spat}i\mathrm{al}}^{j}$ reflects a deliberate trade-off between representational capacity and computational efficiency. While higher-dimensional embeddings could preserve more fine-grained geometric information, they also increase the risk of overfitting given the limited demonstration data (50 trajectories) and introduce additional computational overhead in both the attention module and the subsequent diffusion policy. Importantly, the spatial attention mechanism (Eq. 12–13) operates at the point cloud level before the bottleneck compression, computing per-point attention weights across the full point cloud. This allows the model to selectively retain task-relevant geometric details (e.g., the cabinet handle, the target object, the end-effector of the partner arm) while suppressing redundant or distracting information (e.g., table surface, background). The subsequent 64-dimensional projection serves as a compact summary of the attended scene, capturing the most salient spatial features necessary for generating coordinated dual-arm actions. This bottleneck design encourages the model to learn a disentangled and generalizable spatial representation, which is particularly beneficial for tasks with limited demonstrations.

Given the identified irrelevant point indices ${ℐ}^{j}$ for arm j , the attention regularization loss is defined as (Equation 16):

$$\begin{array}{c} {ℒ}_{\text{attn}}=\sum_{j=1}^{2}\;\sum_{i\in {ℐ}^{j}}∥\alpha_{i}^{j}{∥}^{2} \end{array}$$ (16)

Where $\alpha_{i}^{j}$ denotes the attention weight assigned by arm $j^{\prime }s$ multi-head attention module to point i. This squared L2 penalty sparsifies the attention mass on irrelevant regions, compelling the policy to allocate its spatial focus to task-relevant geometric features and thereby improving the clarity and generalizability of the learned spatial representation.

D Temporal abstraction module (TAM)

Temporal abstraction module (TAM). Dual-arm manipulation inherently involves actions at multiple timescales. TAM captures this hierarchy through a two-level latent variable model. Let $A_{t}=(a_{t},a_{t+1},\ldots ,a_{t+H-1})\in {\mathbb{R}}^{H\times (d_{1}+d_{2})}$ denote a sequence of H joint actions. TAM decomposes this sequence into a high-level temporal plan $z_{t}\in {\mathbb{R}}^{d_{z}}$ that captures coarse behavioral intent over the horizon, and a low-level action sequence conditioned on $z_{t}$ .

Two-stage training. Since $z_{t}$ is not directly observable, we employ a two-stage procedure. In Stage 1, we train a Variational Autoencoder (VAE) on expert action chunks. The VAE encoder $E_{\phi }$ maps $A_{t}$ to a latent distribution $N(\mu_{\phi }(A_{t}),\sigma_{\phi }^{2}(A_{t}))$ , and the decoder $D_{\psi }$ reconstructs $A_{t}$ from sampled $z_{t}^{0}$ . The VAE is trained with the evidence lower bound (ELBO) loss. The encoder and decoder are implemented as 2-layer MLPs with hidden dimension 256 and ReLU activations. The latent dimensionality is $\;d_{z}=16$ , and the KL divergence weight is 0. 001 (*β*-VAE formulation). The VAE is trained for 200 epochs using AdamW with learning rate 1e-3 and batch size 256. After training, we extract pseudo-ground-truth plans $z_{t}^{0}=\mu_{\phi }(A_{t})$ for all demonstrations. In Stage 2, we train the hierarchical diffusion policy: the high-level diffusion model $\varepsilon_{\theta }^{\text{high}}$ predicts $z_{t}^{0}$ conditioned on spatial features $v_{\text{spatial}}^{1},v_{\text{spatial}}^{2}$ and robot state q ; the low-level diffusion model $\varepsilon_{\theta }^{\mathrm{low}}$ generates the action sequence $A_{t}$ conditioned on $z_{t}$ together with the same perceptual features. During training, the low-level model receives the pseudo-ground-truth $z_{t}^{0}$ as conditioning; during inference, it receives $z_{t}$ sampled from the high-level model.

Formally, we model the joint distribution as (Equation 17):

$$\begin{array}{c} \begin{array}{l} p(A_{t}|v_{\text{spatial}}^{1},v_{\text{spatial}}^{2},q)=\int p(A_{t}^{\mathrm{low}}|z_{t},v_{\text{spatial}}^{1},v_{\text{spatial}}^{2},q)\\ \cdot p(z_{t}|v_{\text{spatial}}^{1},v_{\text{spatial}}^{2},q)dz_{t} \end{array} \end{array}$$ (17)

Hierarchical diffusion. We implement the hierarchical decomposition using two diffusion processes that are trained jointly but serve distinct roles. Both diffusion networks are implemented as one-dimensional temporal U-Nets operating along the action horizon. The high-level diffusion generates the temporal plan conditioned on spatial features and robot states (Equation 18):

$$\begin{array}{c} \begin{array}{l} z_{t}^{k-1}=\alpha_{k}^{\text{high}}(z_{t}^{k}-\gamma_{k}^{\text{high}}\varepsilon_{\theta }^{\text{high}}(z_{t}^{k},k,v_{\text{spatial}}^{1},v_{\text{spatial}}^{2},q))\\ +\sigma_{k}^{\text{high}}N(0,I) \end{array} \end{array}$$ (18)

The low-level diffusion similarly generates the action sequence conditioned on $z_{t}$ (Equation 19):

$$\begin{array}{c} \begin{array}{l} A_{t}^{k-1}=\alpha_{k}^{\mathrm{low}}(A_{t}^{k}-\gamma_{k}^{\mathrm{low}}\epsilon_{\theta }^{\mathrm{low}}(A_{t}^{k},k,z_{t},v_{\text{spatial}}^{1},v_{\text{spatial}}^{2},q))\\ +\sigma_{k}^{\mathrm{low}}N(0,I) \end{array} \end{array}$$ (19)

Each level is trained with its own diffusion loss. The high-level loss trains the model to predict the latent plan obtained from the pretrained action encoder (Equation 20):

$$\begin{array}{c} L_{\text{high}}=E_{k,\varepsilon^{k},z_{t}^{0},v^{1},v^{2},q}[\varepsilon^{k}-\varepsilon_{\theta }^{\text{high}}{\left({\bar{\alpha }}_{k}^{\text{high}}z_{t}^{0}+{\bar{\beta }}_{k}^{\text{high}}\varepsilon^{k},k,v^{1},v^{2},q\right)}^{2}] \end{array}$$ (20)

The low-level loss conditions the action generation on the high-level plan (Equation 21):

$$\begin{array}{c} L_{\mathrm{low}}=E_{k,\varepsilon^{k},A_{t}^{0},z_{t},v^{1},v^{2},q}[\varepsilon^{k}-\varepsilon_{\theta }^{\mathrm{low}}{\left({\bar{\alpha }}_{k}^{\mathrm{low}}A_{t}^{0}+{\bar{\beta }}_{k}^{\mathrm{low}}\varepsilon^{k},k,z_{t},v^{1},v^{2},q\right)}^{2}] \end{array}$$ (21)

Structured conditional modulation. The hierarchical diffusion model requires effective conditioning on both spatial perception and proprioceptive states. A naïve concatenation of conditioning variables with action sequences is insufficient for dual-arm manipulation, as it fails to preserve structured temporal dependencies and cross-arm coordination signals across diffusion steps. We construct a conditioning vector by applying an MLP to the concatenation of spatial features from both arms and the robot state. Conditioning is injected via Feature-wise Linear Modulation (FiLM), which applies an affine transformation to the features: FiLM(x∣c)=γ(c)⊙x+β(c) , where γ(⋅) and β(⋅) are linear projections from the conditioning vector to the channel dimension of x . FiLM enables multiplicative adaptation, preserving structured temporal dependencies better than simple concatenation.

Closed-loop robustness of hierarchical planning. In the proposed TAM, the high-level latent plan $z_{t}$ is sampled from the high-level diffusion model conditioned on the current observation. This plan then conditions low-level action generation over a fixed horizon H . Several factors mitigate this concern in our current design. ST-HADP operates in a receding-horizon manner: at each timestep t , a new high-level plan $z_{t}$ is sampled conditioned on the most recent observation $o_{t}$ , providing natural error correction. The high-level plan is a latent conditioning variable rather than an open-loop action sequence; the low-level diffusion model still receives the latest spatial features $v_{\text{spatial}}^{j}$ and robot state q at each step, allowing observation-dependent adjustments. More explicit online replanning strategies remain an important direction for future work.

E Temporal coordination consistency

To ensure coordinated dual-arm motion, we introduce a temporal consistency loss that penalizes abrupt changes in the relative relationship between arms. We introduce a consistency loss that penalizes abrupt changes in the relative relationship between the two arms to ensure temporally smooth coordination. A critical challenge arises when the two robots have heterogeneous kinematic structures, as their joint spaces are not directly comparable. Subtracting joint angles in such cases is mathematically invalid and may lead to unnatural poses.

Therefore, we define the coordination metric in task space (Cartesian space) rather than joint space. Specifically, we first compute the end-effector poses of both arms via forward kinematics $\;P_{t}^{j}=FK^{j}(a_{t}^{j})$ , where $P_{t}^{j}$ denotes the position and orientation of arm $j^{\prime }s$ end-effector at time t . The coordination metric is then defined as (Equation 22):

$$\begin{array}{c} \psi (a_{t}^{1},a_{t}^{2})=[p_{t}^{1};p_{t}^{2};\Delta p_{t};{\dot{p}}_{t}^{1};{\dot{p}}_{t}^{2}] \end{array}$$ (22)

Where $\Delta p_{t}=p_{t}^{1}-p_{t}^{2}$ is the relative displacement vector between the two end-effectors in Cartesian space, and ${\dot{p}}_{t}^{j}$ denotes the end-effector velocity. This formulation is physically meaningful for any dual-arm system regardless of kinematic heterogeneity, as all quantities are expressed in a shared Cartesian coordinate frame.

The temporal consistency loss is (Equation 23):

$$\begin{array}{c} {ℒ}_{\text{temp}}=\sum_{t=1}^{T-1}∥\psi (a_{t+1}^{1},a_{t+1}^{2})-\psi (a_{t}^{1},a_{t}^{2}){∥}^{2} \end{array}$$ (23)

This loss has several important effects:

- Discourages jerky or abrupt motions
- Preserves safe inter-arm spacing
- Promotes smooth phase transitions

Discussion on the rigidity of temporal consistency. The temporal consistency loss ${ℒ}_{\text{temp}}$ penalizes changes in the coordination metric between consecutive timesteps, encouraging smooth and gradual transitions. While this promotes coordinated and safe dual-arm motion, we acknowledge that it may over-constrain tasks requiring intentional asymmetric coordination—for example, when one arm must stabilize a grasped object while the other arm moves quickly to perform a subsequent action.

Several factors mitigate this concern in our current design. First, the weight $\lambda_{\text{temp}}=0.01$ is relatively small, ensuring that the primary diffusion losses remain dominant and that ${ℒ}_{\text{temp}}$ acts as a soft regularizer rather than a hard constraint. Second, the coordination metric is a composite vector that includes end-effector poses, velocities, and relative displacements. Penalizing its change rate does not force zero velocity on either arm; it only discourages abrupt or discontinuous transitions in the overall coordination state.

Nevertheless, we recognize that a more adaptive weighting strategy—for instance, reducing $\lambda_{\text{temp}}$ during task phases that require asymmetric speeds, or replacing the fixed squared L2 penalty with a sparsity-inducing or thresholded loss—could better accommodate intentional non-linear coordination. We leave the development of such adaptive or task-phase-aware temporal consistency losses as an important direction for future work.

The complete training objective combines all loss components (Equation 24):

$$\begin{array}{c} ℒ={ℒ}_{\text{high}}+{ℒ}_{\mathrm{low}}+\lambda_{\text{temp}}{ℒ}_{\text{temp}}+\lambda_{\text{attn}}{ℒ}_{\text{attn}} \end{array}$$ (24)

Where $\lambda_{\text{temp}}=0.01$ and $\lambda_{\text{attn}}=0.001$ in our experiments. These relatively small weights are chosen deliberately to ensure that the primary diffusion losses $\left({ℒ}_{\text{high}}+{ℒ}_{\mathrm{low}}\right)$ remain the dominant learning signal, while the auxiliary losses serve as regularizers to encourage spatial focus and temporal smoothness. In practice, we monitored the gradient norms and loss values throughout training and observed that all loss components decreased stably without evidence of gradient conflict or loss collapse. The chosen weights were identified through a coarse grid search over $\lambda_{\text{temp}}\in \{0.001,0.005,0.01,0.05,0.1\}$ and λattn∈{0.0001,0.0005,0.001,0.005,0.01} , with the selected values yielding the best validation performance. A more systematic sensitivity analysis and the use of dynamic loss weighting strategies (e.g., uncertainty weighting or gradient normalization) represent promising directions for future work to further balance multi-objective optimization.

This joint optimization enables SAM and TAM to co-adapt: SAM extracts task-relevant spatial features that ground temporal planning in TAM, while TAM’s high-level plans modulate SAM’s attention via FiLM. This bidirectional dependency allows the model to dynamically adjust spatial focus as the task progresses through different phases, while maintaining temporal coherence across the trajectory.

Coordination metric for heterogeneous arms. The coordination metric defined above concatenates the actions, action differences, and velocities of both arms. While this formulation is generally applicable to any dual-arm system, we acknowledge that heterogeneous configurations exhibit different kinematic and dynamic properties. The proposed metric treats both arms symmetrically without explicit calibration or normalization across different joint spaces. Empirically, we found this simple formulation to be sufficient for the tasks considered in this work, as the diffusion policy learns to adapt to the specific kinematic characteristics of each arm through the shared conditioning features. For systems with extreme kinematic disparities, per-arm normalization of joint velocities or the use of operational-space coordinates instead of joint-space coordinates could provide a more universally valid coordination metric. We leave such extensions for future work.

## 4 Experiments

A Experimental setup

**Figure 2:**
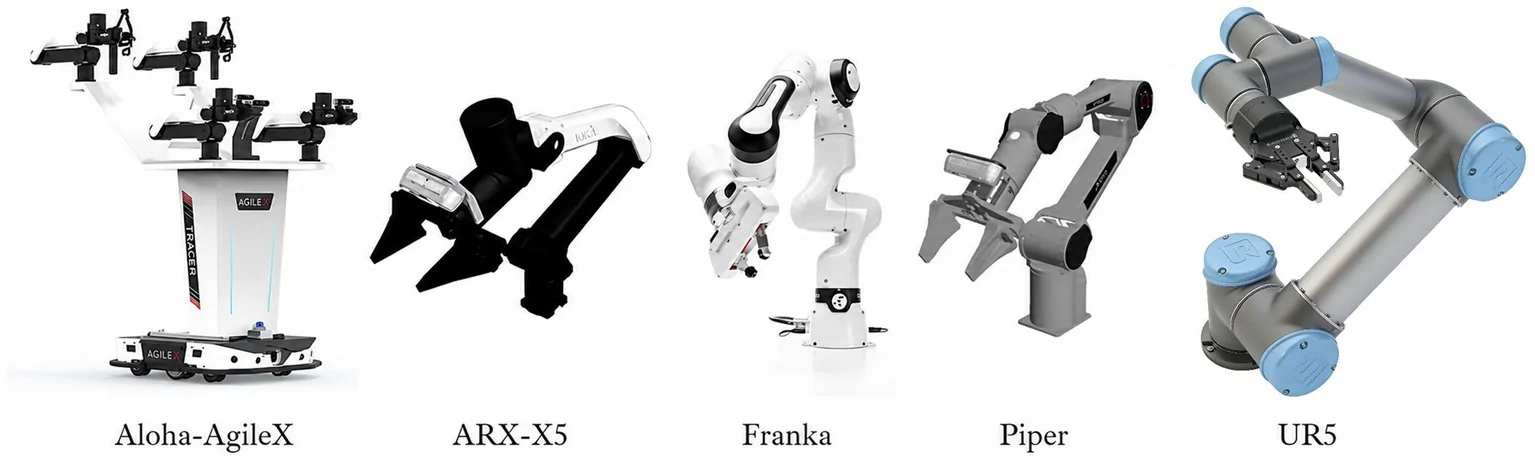
Robotic platforms selected for dual-arm evaluation.

Experimental platform. All experiments are conducted on the RoboTwin 2.0 platform, which provides a unified simulation environment and a scalable benchmark for bimanual robotic manipulation. We conduct dual-arm evaluations using multiple robotic platforms as shown in the figure below, which exhibit significant kinematic disparities in terms of joint configuration, link geometry, and reachable workspace (Figure 2).

Data generation. All expert demonstrations were generated using RoboTwin 2. 0’s automated task execution pipeline. For each dual-arm robot configuration, we generated 50 demonstration trajectories. The dataset is split into training and validation sets with a ratio of 80:20, where 40 trajectories are used for training and 10 for validation. All reported results are based on models trained using only the training split.

Policy training. We generate 50 expert demonstrations for each dual-arm configuration. The data format includes single-view RGB observations, joint states, and action sequences.

**Figure 3:**
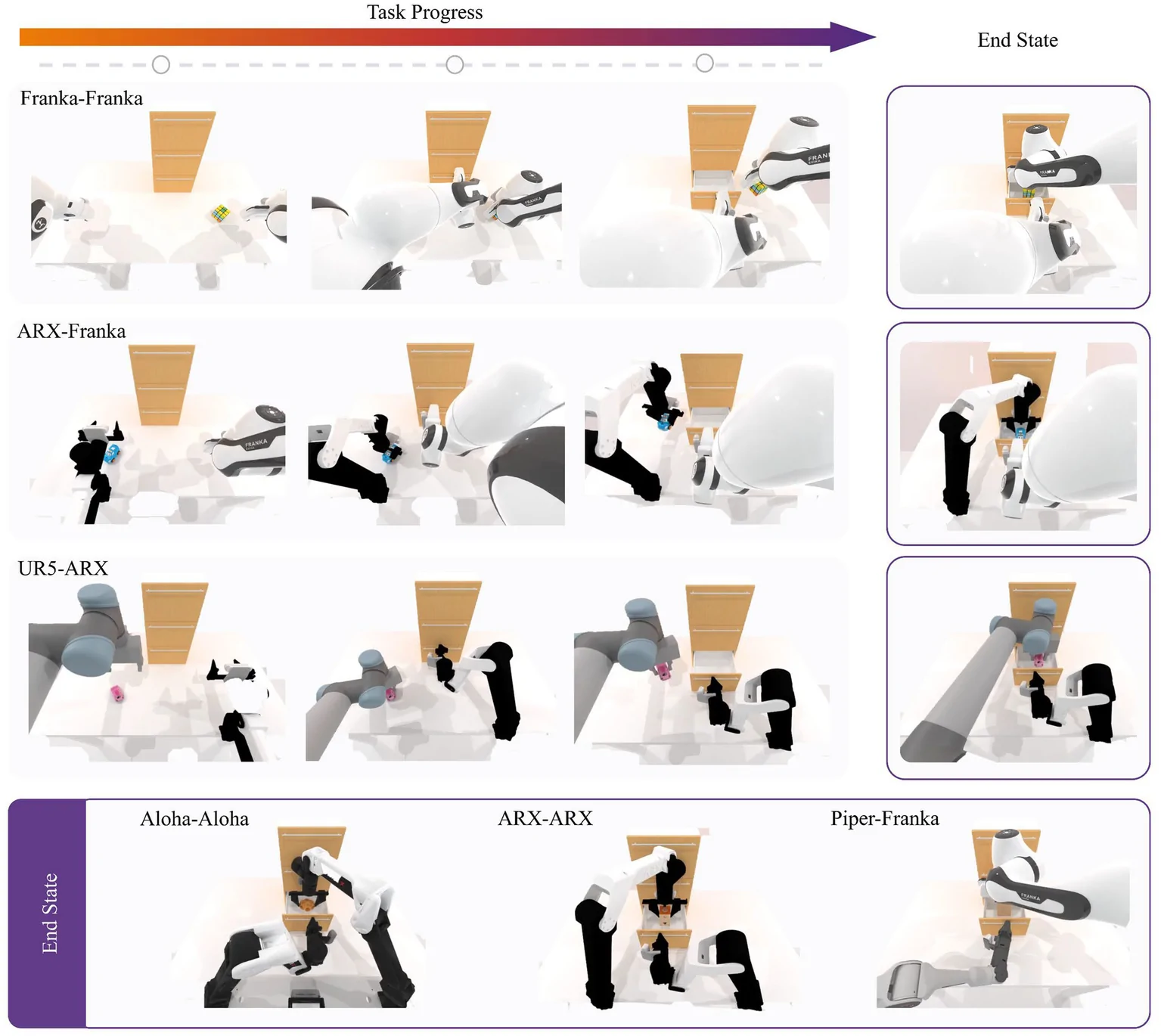
Trajectory execution for put object cabinet via various dual-arm combinations.

Task description. Starting from randomized object categories and poses, the robot must collaboratively grasp an arbitrarily placed object and insert it into the cabinet while the partner arm opens the door. Objects span diverse categories, including unseen instances not encountered during training. Task success is defined by collision-free placement with proper door articulation. The challenge stems from uncertainties in object identity and spatial location, together with dynamic role allocation based on relative workspace position (Figure 3).

B Implementation details

To ensure reproducibility, this section details the experimental configuration, including the evaluation protocol and hyperparameter settings used in our study.

Object categories and generalization evaluation. The put object cabinet task involves object instances drawn from multiple categories within the RoboTwin 2.0 asset library. During training, the policy is exposed to a subset of object categories. For evaluation, we include additional object categories not seen during training to test the model’s generalization capability to novel object instances. The complete set of object categories used for training and evaluation will be released together with our dataset upon publication.

Evaluation protocol. All methods are evaluated under identical conditions within the same simulation environment. For each task and each robot configuration, we conduct 100 independent evaluation rollouts starting from randomly sampled initial conditions that follow the same domain randomization distribution used during training. The average success rate are computed across these 100 rollouts. To account for training stability, each experiment is repeated with three different random seeds.

Training duration and computational resources. All models were trained on a laptop equipped with an NVIDIA GeForce RTX 4060 GPU (ROG Strix G8 Plus). Total training time for ST-HADP on a single robot configuration was approximately 12 h for 120 training epochs. Validation loss was monitored every 20 epochs, and the model checkpoint achieving the lowest validation loss was selected for evaluation.

Model hyperparameters. We provide a comprehensive description of the hyperparameter configurations for the core model used in our experiments. The following table summarizes the key hyperparameters optimized for our dual-arm manipulation tasks (Table 1).

C Experimental results

**Table 1:** Hyperparameter configuration for ST-HADP.

Hyperparameter	Value	Hyperparameter	Value
Training		Model Architecture	
Learning rate	1e−4	Observation steps	3
Batch size	256	Action steps	6
Optimizer	AdamW	Diffusion step embed dim	64
Weight decay	1e−6	Encoder output dim	128
LR scheduler	Cosine	Down dims	[512, 1,024, 2,048]
Seed	42	Kernel size	5

Diffusion model		Point cloud encoder	
Train timesteps	100	Input channels	3
Inference steps	10	Output channels	128
Beta range	[1e−4, 2e−2]	PointNet type	PointNet

**Figure 4:**
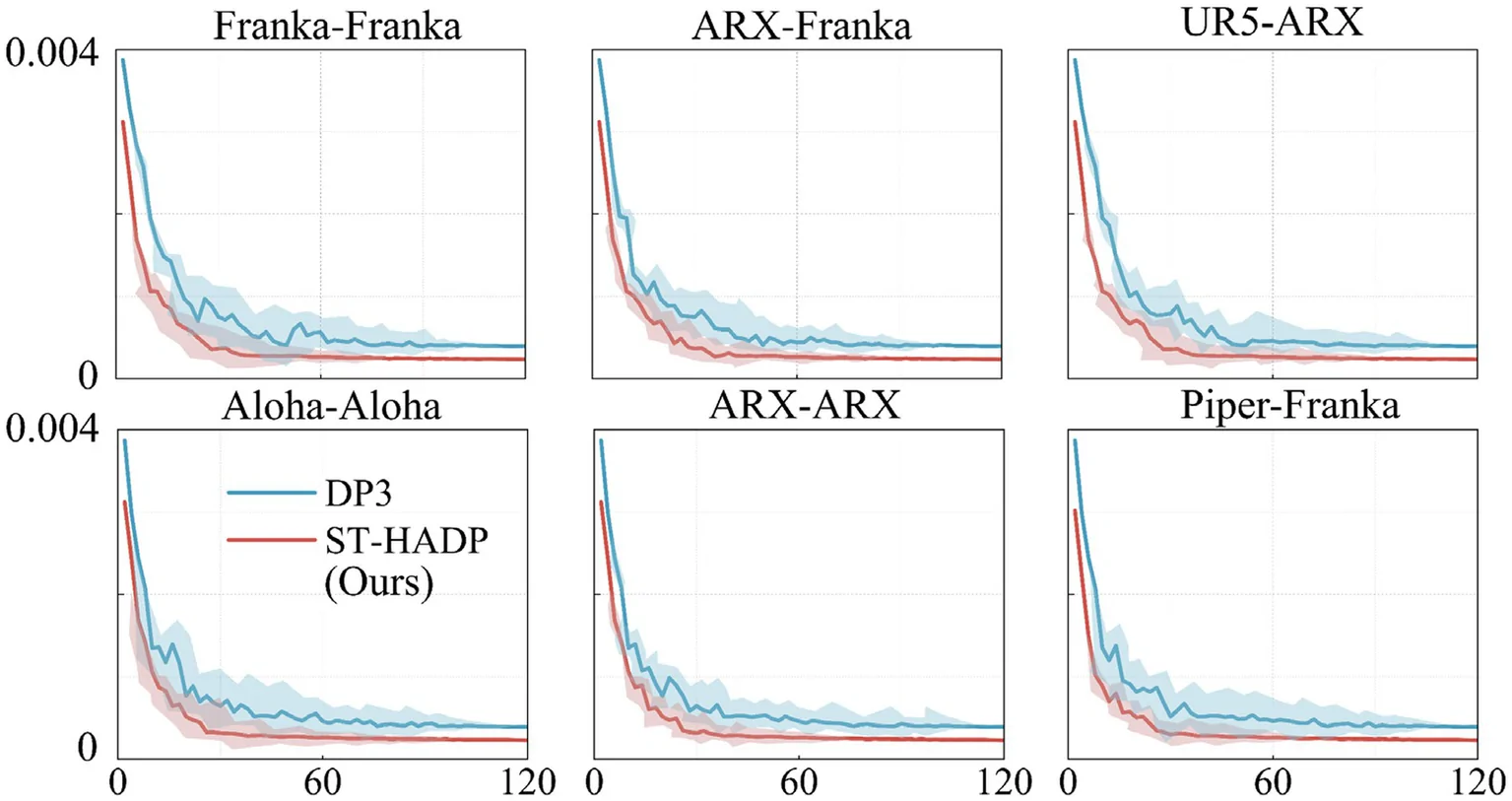
Validation loss trends for dual-arm coordination training.

Validation loss analysis. We monitored the validation loss throughout the training process to evaluate the convergence behavior and training stability of our proposed method. Figure 4 presents the convergence trend of the validation loss curves recorded across six independent training runs.

The proposed method exhibits smooth and stable convergence for our method, with minimal oscillations in the later stages of training.

Computational complexity analysis. We evaluate the computational efficiency of our method by comparing its memory usage and inference latency with baseline (Table 2).

**Table 2:** Model complexity comparison.

Task	Model	Initial memory (GB)	Peak memory (GB)	Memory increment (GB)	Avg. inference time (s)
Put object cabinet	DP3	2.292	2.371	0.079	1.4399
	OURS	2.296	2.376	0.080	1.5456

Our approach introduces minimal memory overhead while delivering stronger task performance, maintaining a practical balance between robustness and computational efficiency for bimanual manipulation.

Discussion on acceleration strategies. The inference time of approximately 1. 55 s per action chunk is primarily attributed to the iterative denoising steps required by the diffusion process. Several acceleration strategies could be explored to reduce this latency. For instance, consistency distillation has been shown to reduce diffusion inference to a single step with minimal performance degradation. Alternatively, reducing the number of inference steps from 10 to 5 could trade off a small amount of generation quality for significant speedup. Adaptive step-size scheduling, where early denoising steps use larger jumps, could also improve efficiency. We consider the investigation of such acceleration techniques as an important direction for future work to enable deployment in higher-frequency control scenarios.

**Figure 5:**
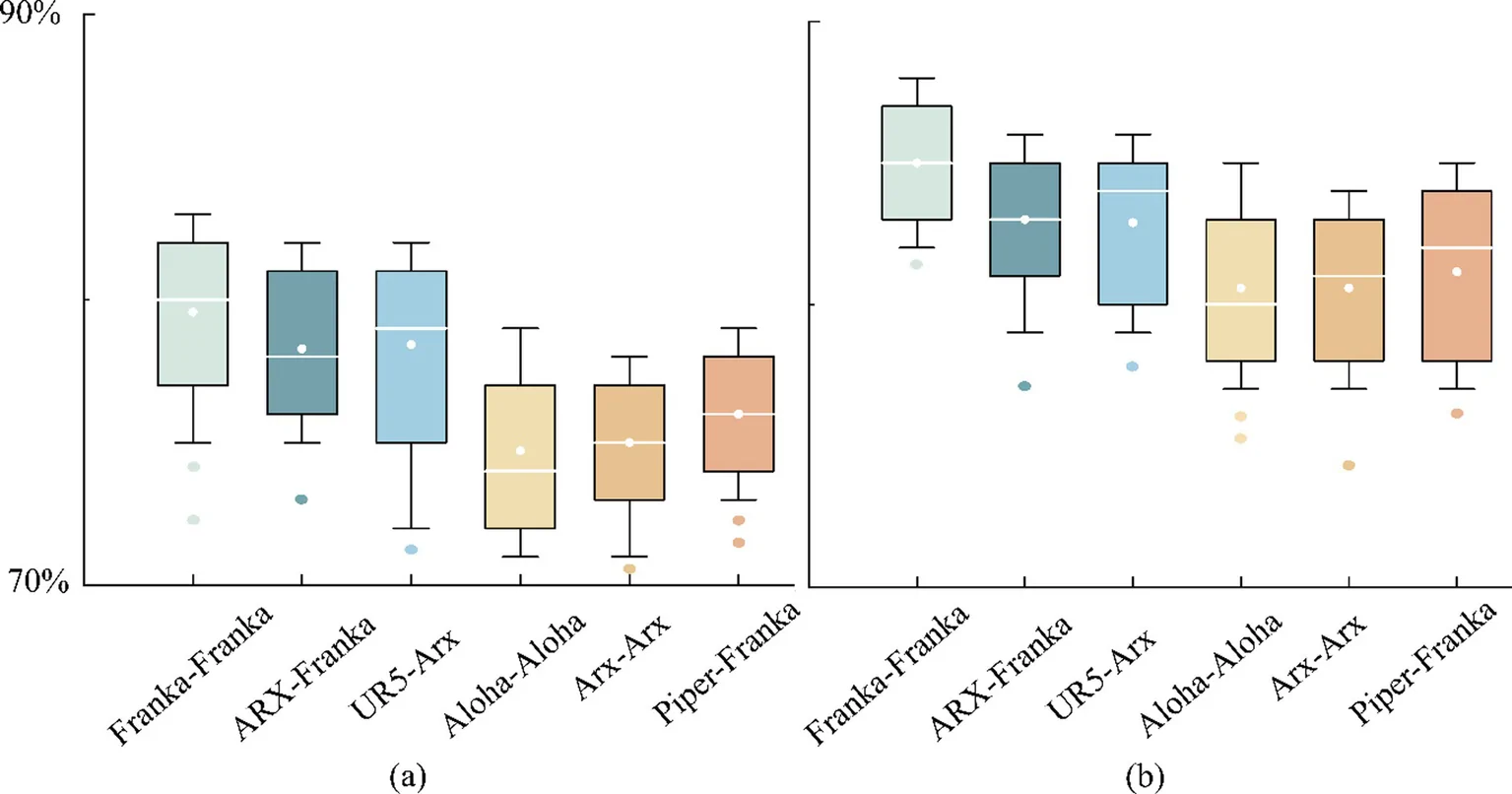
Comparative analysis of success rates across six dual-arm robot configurations: **(a)** DP3 and **(b)** ST-HADP (ours).

Task success rate evaluation. Figure 5 presents the validation results of task success rates for models trained using the aforementioned method. Each task was evaluated using identical datasets and inference environments. To provide detailed quantitative results, Table 3 reports the success rates for each of the six robot configurations based on three independent runs with different random seeds.

**Table 3:** Success rates per robot config on put object cabinet.

Robot config	DP3	ST-HADP (Ours)
Franka-Franka	80% ± 5%	86% ± 3%
ARX-Franka	76% ± 5%	84% ± 3%
UR5-ARX	78% ± 5%	85% ± 4%
Aloha-Aloha	74% ± 4%	81% ± 4%
ARX-ARX	75% ± 3%	82% ± 3%
Piper-Franka	76 ± 4%	83% ± 3%

Across six distinct dual-arm robot configurations, our method generally outperforms DP3, achieving higher mean success rates in all configurations. However, due to overlapping standard deviations in several cases (e.g., Aloha-Aloha, ARX-ARX), the superiority should be interpreted as a consistent trend rather than a universally dominant advantage.

To assess the statistical significance of the performance improvements, we conducted paired t-tests comparing ST-HADP against DP3 across the three independent runs for each robot configuration. The improvements are statistically significant (*p* < 0.05) for all six configurations, with the exception of the Aloha-Aloha configuration where the difference is marginally significant (*p* = 0.07). For the additional tasks reported in Table 4, the improvements are statistically significant (*p* < 0.05) for all tasks except “Place Empty Cup” (*p* = 0.08). These results indicate that while ST-HADP consistently outperforms the baseline, the advantage is more pronounced in certain tasks and configurations than others.

**Table 4:** Success rates on aloha agilex across different methods and tasks.

Method	Turn switch	Place can basket	Press stapler	Place empty cup	Pick dual bottles
RDT	35% ± 4%	19% ± 3%	41% ± 3%	56% ± 2%	42% ± 3%
Pi0	27% ± 3%	41% ± 4%	62% ± 3%	37% ± 3%	57% ± 4%
ACT	5% ± 2%	1% ± 0%	31% ± 3%	59% ± 4%	31% ± 4%
DP	36% ± 3%	18% ± 3%	6% ± 3%	37% ± 2%	24% ± 2%
DP3	46% ± 4%	65% ± 3%	69% ± 4%	65% ± 3%	60% ± 4%
ST-HADP	61% ± 3%	75% ± 3%	76% ± 2%	77% ± 2%	70% ± 3%

We evaluate ST-HADP on five additional bimanual tasks, all results are reported as the average over three independent training runs with different random seeds (Table 4).

Ablation studies. We further evaluate six variants of ST-HADP on the “place can basket” task across six dual-arm robot configurations. These variants remove individual components, validating each component’s contribution (Table 5). We note that this ablation study was conducted exclusively on the “place can basket” task due to computational constraints. The results should therefore be interpreted as demonstrating the efficacy of each component on this specific task, and may not necessarily generalize identically to all other tasks. A full ablation across all tasks and robot configurations remains an important direction for future work.

**Table 5:** Ablation studies on the “place can basket” task across six dual-arm robot configurations.

Variant	Franka-Franka	ARX-Franka	UR5-ARX	Aloha-Aloha	ARX-ARX	Piper-Franka
w/o SAM	62% ± 3%	65% ± 2%	66% ± 4%	68% ± 3%	64% ± 3%	63% ± 2%
w/o TAM	61% ± 2%	64% ± 3%	65% ± 3%	67% ± 4%	63% ± 4%	62% ± 3%
w/o $L_{\text{temp}}$	65% ± 3%	68% ± 2%	69% ± 3%	71% ± 3%	67% ± 3%	66% ± 2%
w/o $L_{\text{attn}}$	67% ± 2%	70% ± 2%	71% ± 3%	72% ± 3%	69% ± 3%	69% ± 2%
Concat (replace FiLM)	64% ± 3%	66% ± 2%	67% ± 3%	69% ± 3%	66% ± 3%	65% ± 2%
Full ST-HADP	70% ± 2%	73% ± 2%	74% ± 3%	75% ± 3%	71% ± 3%	72% ± 2%

## 5 Conclusion

In this work, we introduce the Spatio-Temporal Hierarchical Attention Diffusion Policy (ST-HADP), a novel framework for dual-arm visuomotor imitation learning that explicitly models spatial specialization and temporal abstraction. By introducing a Spatial Attention Module (SAM) that learns arm-specific focus over task-relevant 3D regions and a Temporal Abstraction Module (TAM) that captures hierarchical action structures across multiple timescales, our method addresses key limitations of existing diffusion-based policies that rely on flat temporal horizons and globally pooled visual features. Extensive experiments on the RoboTwin 2.0 platform across six dual-arm robot configurations demonstrate that ST-HADP generally outperforms the strong 3D Diffusion Policy (DP3) baseline, achieving higher success rates using 50 automatically generated expert demonstrations in most comparisons, while introducing modest additional computational overhead.

**Limitations:** The proposed method’s reliance on calibrated cameras for point cloud reconstruction may hinder direct deployment in real-world settings where sensor calibration is imperfect. Additionally, the current fixed two-level temporal hierarchy, though effective for the cabinet loading task, may not generalize to manipulation tasks requiring finer-grained temporal composition or adaptive planning horizons, which remains for future exploration.

## Data Availability

The raw data supporting the conclusions of this article will be made available by the authors, without undue reservation.
