# Correction: ST-HADP: Spatio-Temporal hierarchical attention diffusion policy for long-horizon generalizable bimanual visuomotor imitation

**DOI:** 10.3389/fnbot.2026.1959872

**Published:** 2026-08-28

**Authors:** Xukun Liu, Fengjuan Xie, Shibo Liu, Xu Sun, Zhenyu Liu, Guangning Li, Shenggang Wei

**Affiliations:** Northwest Institute of Mechanical and Electrical Engineering, Xianyang, Shaanxi, China

**Keywords:** 3D visuomotor policy, bimanual manipulation, hierarchical diffusion, imitation learning, Spatio-Temporal hierarchical attention

There was a mistake in the figures and tables as published. During the proofreading stage, we explicitly requested that the font sizes within the figures be kept slightly smaller than that of the main text, in line with our original manuscript. However, this typesetting instruction was not carried out during the final production process, which compromised the clarity and readability of the data. To address both issues, we have now revised the figure layouts alongside upgrading the resolution, ensuring proper data clarity and typographical consistency.

The corrected Figures 1–5 appear below.

**Figure 1:**
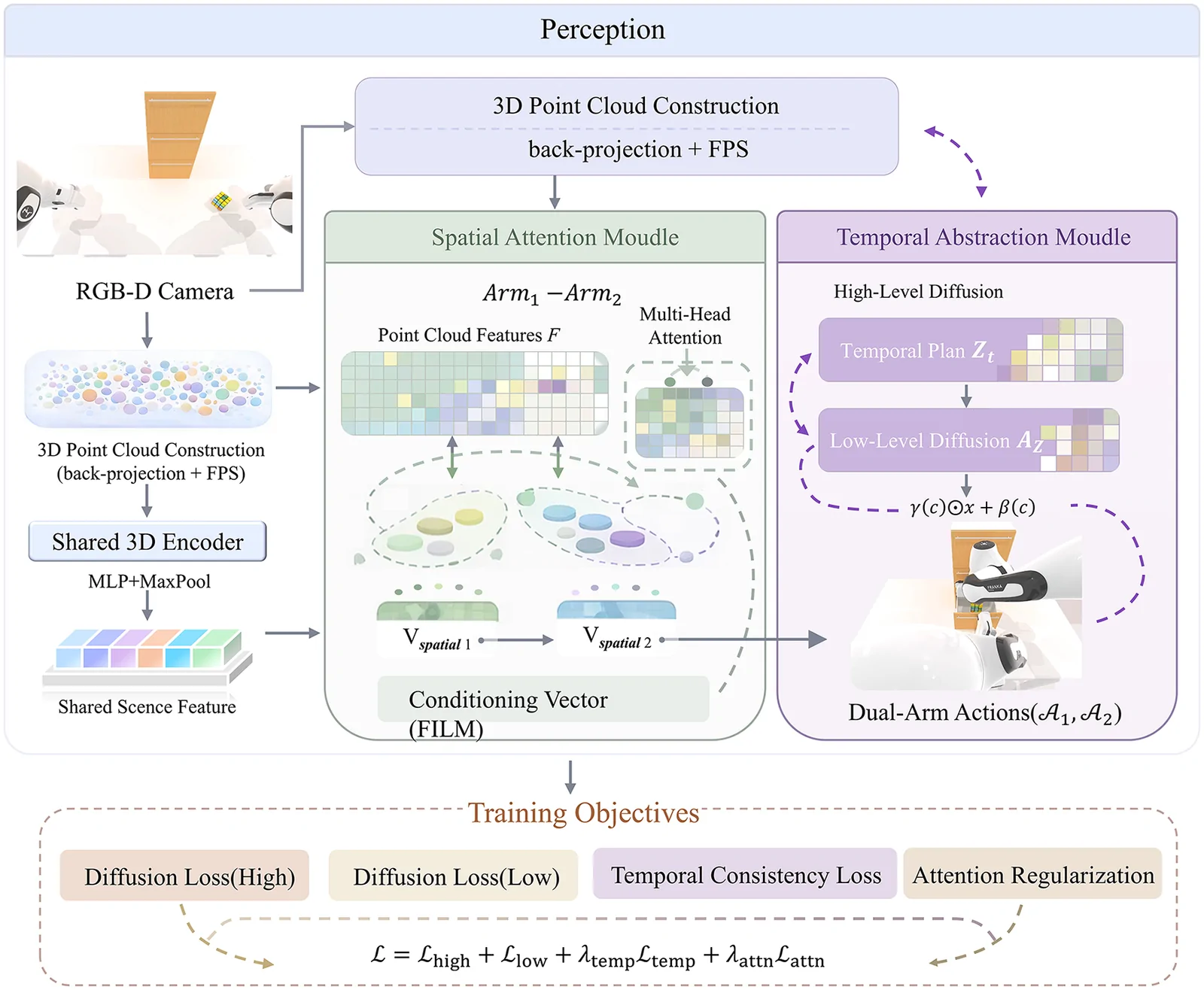
Overview of ST-HADP architecture.

**Figure 2:**
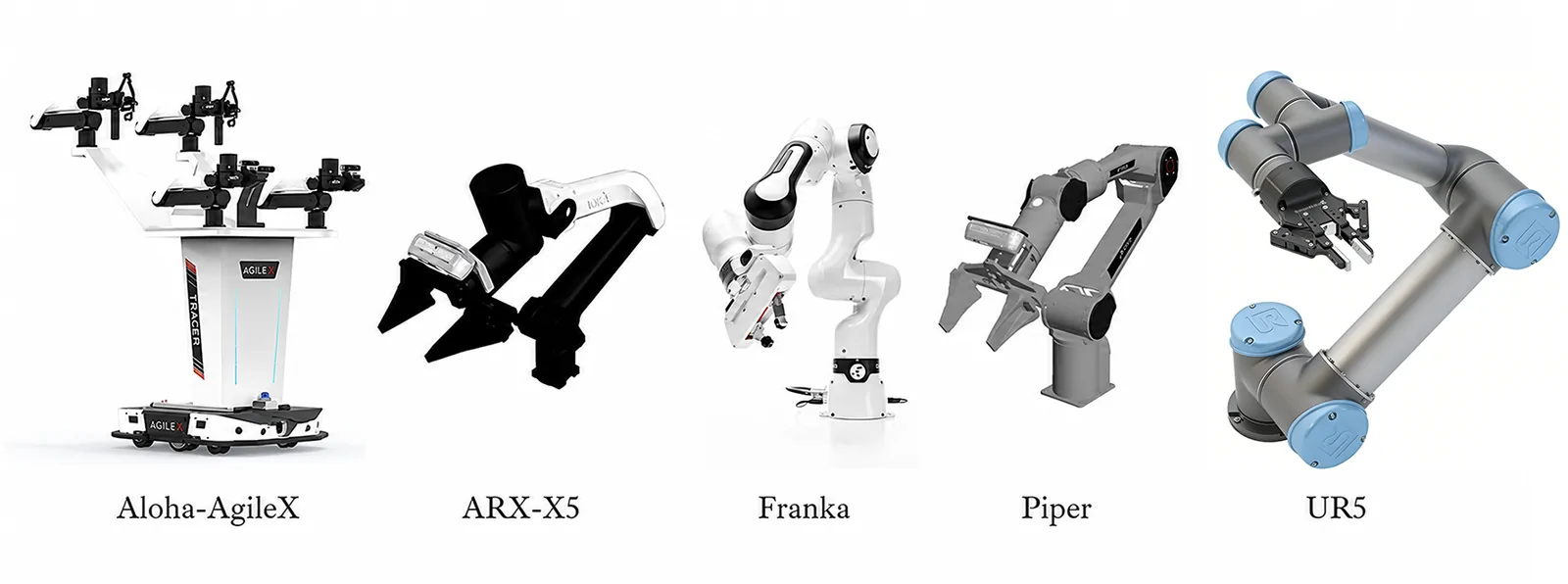
Robotic platforms selected for dual-arm evaluation.

**Figure 3:**
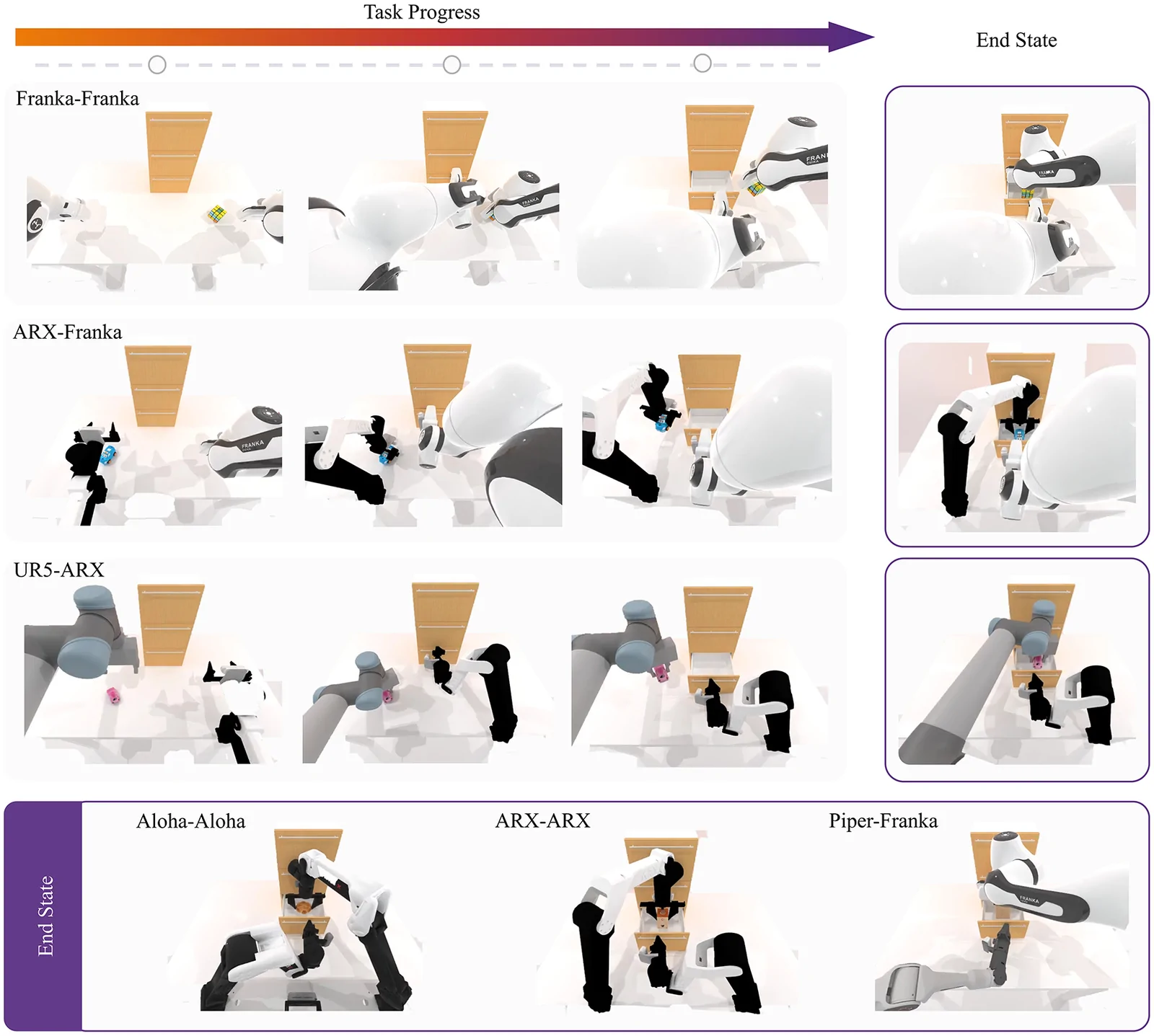
Trajectory execution for put object cabinet via various dual-arm combinations.

**Figure 4:**
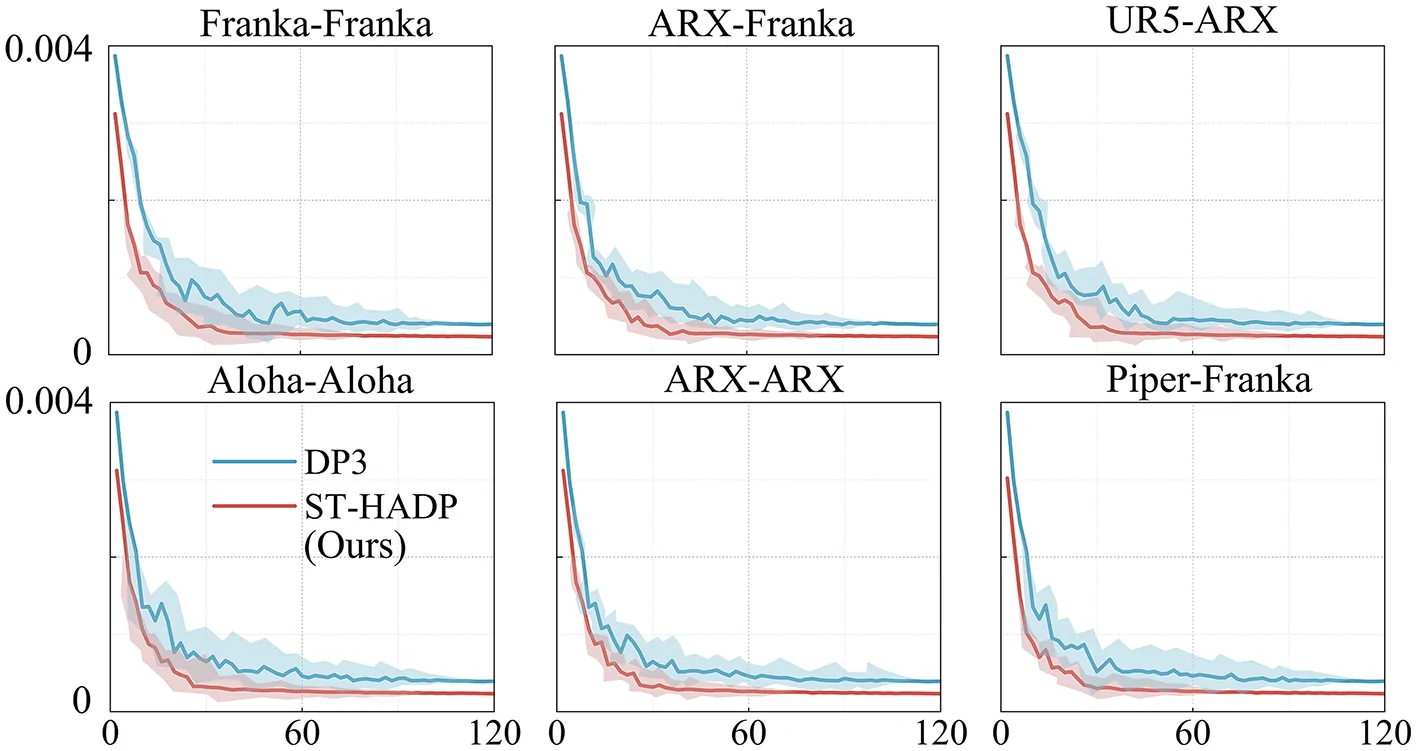
Validation loss trends for dual-arm coordination training.

**Figure 5:**
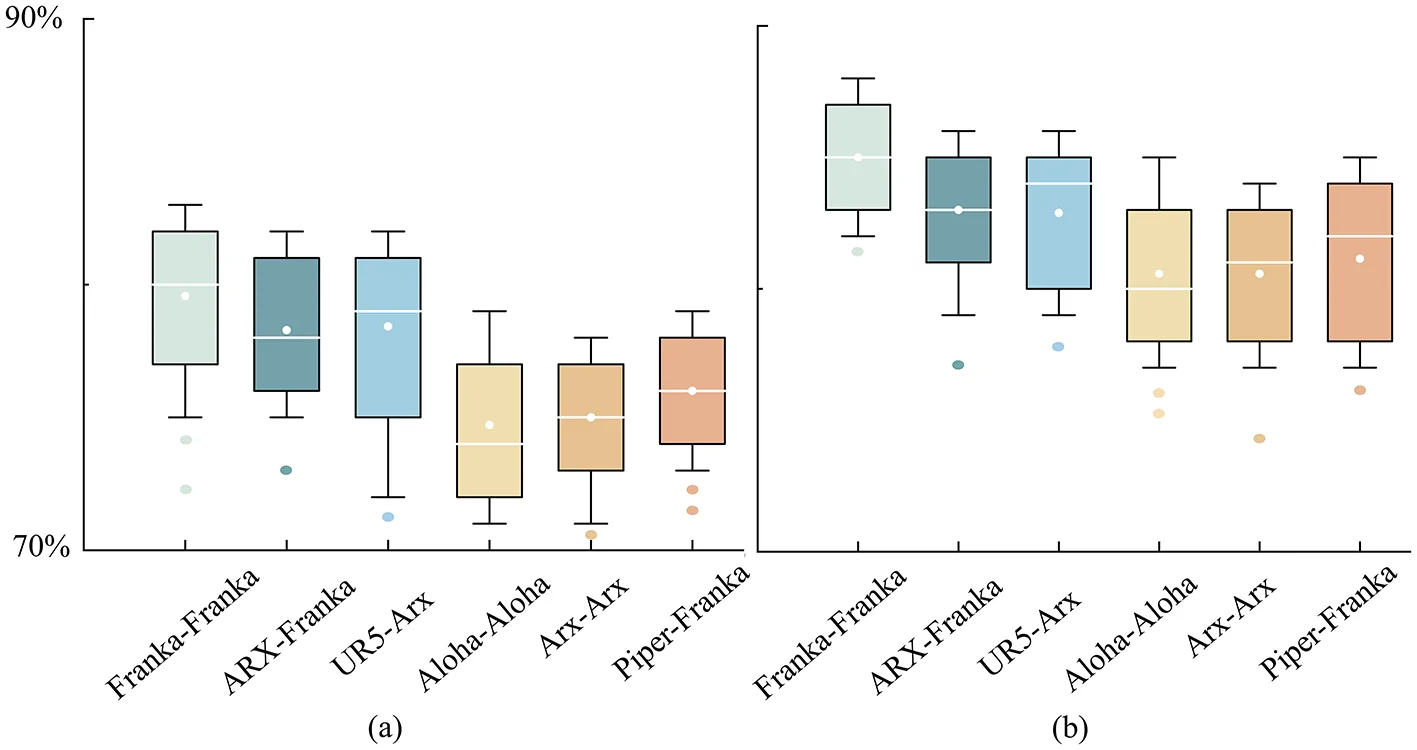
Comparative analysis of success rates across six dual-arm robot configurations: **(a)** DP3 and **(b)** ST-HADP (ours).

Additionally, in the Tables 1, 2, 4, 5, certain content was not centered as per the author correction version approved during the proofing stage, which deviated from the intended formatting.

**Table 1:** Hyperparameter configuration for ST-HADP.

Hyperparameter	Value	Hyperparameter	Value
Training		Model Architecture	
Learning rate	1e-4	Observation steps	3
Batch size	256	Action steps	6
Optimizer	AdamW	Diffusion step embed dim	64
Weight decay	1e-6	Encoder output dim	128
LR scheduler	Cosine	Down dims	[512, 1,024, 2,048]
Seed	42	Kernel size	5
Diffusion model		Point cloud encoder	
Train timesteps	100	Input channels	3
Inference steps	10	Output channels	128
Beta range	[1e-4, 2e-2]	PointNet type	Pointnet

**Table 2:** Model complexity comparison.

Task	Model	Initial memory(GB)	Peak memory(GB)	Memory increment(GB)	Avg. inference time (s)
Put object cabinet	DP3	2.292	2.371	0.079	1.4399
	OURS	2.296	2.376	0.080	1.5456

**Table 4:** Success rates on Aloha Agilex across different methods and tasks.

Method	Turn switch	Place can basket	Press stapler	Place empty cup	Pick dual bottles
RDT	35% ± 4%	19% ± 3%	41% ± 3%	56% ± 2%	42% ± 3%
Pi0	27% ± 3%	41% ± 4%	62% ± 3%	37% ± 3%	57% ± 4%
ACT	5% ± 2%	1% ± 0%	31% ± 3%	59% ± 4%	31% ± 4%
DP	36% ± 3%	18% ± 3%	6% ± 3%	37% ± 2%	24% ± 2%
DP3	46% ± 4%	65% ± 3%	69% ± 4%	65% ± 3%	60% ± 4%
ST-HADP	61% ± 3%	75% ± 3%	76% ± 2%	77% ± 2%	70% ± 3%

**Table 5:** Ablation studies on the “place can basket” task across six dual-arm robot configurations.

Variant	Franka-Franka	ARX-Franka	UR5-ARX	Aloha-Aloha	ARX-ARX	Piper-Franka
w/o SAM	62% ± 3%	65% ± 2%	66% ± 4%	68% ± 3%	64% ± 3%	63% ± 2%
w/o TAM	61% ± 2%	64% ± 3%	65% ± 3%	67% ± 4%	63% ± 4%	62% ± 3%
w/o $L_{temp}$	65% ± 3%	68% ± 2%	69% ± 3%	71% ± 3%	67% ± 3%	66% ± 2%
w/o $L_{attn}$	67% ± 2%	70% ± 2%	71% ± 3%	72% ± 3%	69% ± 3%	69% ± 2%
Concat (replace FiLM)	64% ± 3%	66% ± 2%	67% ± 3%	69% ± 3%	66% ± 3%	65% ± 2%
Full ST-HADP	70% ± 2%	73% ± 2%	74% ± 3%	75% ± 3%	71% ± 3%	72% ± 2%

The original version of this article has been updated.

## Generative AI statement

Any alternative text (alt text) provided alongside figures in this article has been generated by Frontiers with the support of artificial intelligence and reasonable efforts have been made to ensure accuracy, including review by the authors wherever possible. If you identify any issues, please contact us.

